# Can artificial intelligence manage malnutrition? A critical look at ChatGPT's performance in geriatric nutrition

**DOI:** 10.1590/1806-9282.20252286

**Published:** 2026-06-29

**Authors:** Ahmet Cigiloglu, Eyyup Murat Efendioglu, Zeynel Abidin Ozturk

**Affiliations:** 1 Kahramanmaraş Necip Fazıl City Hospital, Department of Internal Medicine, Division of Geriatric Medicine – Kahramanmaraş, Turkey.; 2 Gaziantep City Hospital, Department of Internal Medicine, Division of Geriatric Medicine – Gaziantep, Turkey.; 3 Gaziantep University, Faculty of Medicine, Department of Internal Medicine, Division of Geriatric Medicine – Gaziantep, Turkey.

**Keywords:** Artificial intelligence, ChatGPT, Malnutrition, Older adults, Accuracy

## Abstract

**BACKGROUND:**

Artificial intelligence represents a rapidly advancing innovation in healthcare with the potential to revolutionize the field of clinical nutrition.

**OBJECTIVE:**

The aim of this study was to evaluate ChatGPT's potential to support the clinical decision-making process regarding nutrition in older adults.

**METHODS:**

Twelve questions and three clinical vignettes addressing fundamental concepts of malnutrition, including general information, diagnosis, follow-up, and treatment, were created and asked to ChatGPT. Three geriatricians independently examined ChatGPT's responses. The quality of the responses was assessed using the Quality Analysis of Medical Artificial Intelligence tool.

**RESULTS:**

The inter-rater reliability among the authors was calculated, and an excellent intraclass correlation coefficient of 0.84 (95%CI 0.77–0.89; p<0.001) was found. The total mean Quality Analysis of Medical Artificial Intelligence score for the ChatGPT-generated responses to questions related to malnutrition was 26.60, indicating very good quality. In evaluating the clinical scenarios, the lowest scores were observed in source use. The total Quality Analysis of Medical Artificial Intelligence, accuracy, relevance, and use of sources scores for the clinical scenario involving the patient with a hip fracture were statistically significantly lower compared to other scenarios.

**CONCLUSION:**

Our study highlighted that ChatGPT has the potential to generate correct answers related to complex clinical scenarios about malnutrition. ChatGPT can help clinicians make more informed decisions regarding patients’ nutritional requirements and management by utilizing more up-to-date medical resources and guidelines.

## INTRODUCTION

Artificial intelligence (AI) and large language models (LLMs) in particular are increasingly being used in healthcare. In healthcare delivery, the contribution of AI in enhancing clinical decision-making has garnered the most attention, particularly concerning prognostic assessment, diagnostic accuracy, treatment, clinician workflow optimization, and expansion of clinical expertise^1^. AI has demonstrated its potential in enhancing the quality of medical documentation and synthesizing information for clinicians. These advancements can alleviate the cognitive burden on healthcare practitioners and rectify the deficiencies inherent in human-generated documentation^2^. By integrating and analyzing diverse data types such as electronic health records, genomic information, and imaging results, LLMs are increasingly positioned to support clinical decision-making in treatment selection^3^. An increasing number of studies are being conducted on the use of AI in various fields of geriatric medicine. A study evaluating responses provided by AI to questions related to geriatric medicine revealed that scores varied significantly depending on the area of knowledge, with responses concerning the diagnosis/performing of complementary tests receiving the lowest scores^4^.

Malnutrition is an increasingly recognized geriatric syndrome associated with morbidity, mortality, and increased costs of care. Improving early recognition and treatment of malnutrition is crucial. Many cases of malnutrition go unnoticed, which leads to further complications and increases mortality. A meta-analysis has shown that the prevalence of malnutrition and the risk of malnutrition in older adults with dementia reach approximately 80%^5^. In certain conditions commonly seen in older adults, nutritional assessment should be prioritized. In cases with hip fractures and pressure ulcers, adequate and early nutritional support is crucial to promote rapid recovery, prevent complications and ensure independence, even if the patient is not malnourished^6,7^.

A recent review has shown that AI algorithms could identify new relationships between diet and disease outcomes, enabling clinicians to make evidence-based nutrition recommendations^8^. In another study, researchers developed the Malnutrition Universal Screening Tool (MUST)-Plus, a machine learning–based screening tool, which markedly enhanced the early identification and documentation of malnutrition and was well accepted by registered dietitians^9^. ChatGPT has strong capabilities in nutritional evaluation by estimating caloric requirements and recommending nutrient-dense foods, as well as in identifying nutritional issues using technical terminology^10^.

One of the primary roles of AI is to enhance clinical decision-making and streamline the delivery of healthcare services^11^. However, there is little evidence on the quality, accuracy, applicability, ethical challenges, and safety of these applications^12^. Therefore, we assessed the extent to which ChatGPT provided accurate nutritional advice among older adults in the present study.

## METHODS

**Figure 1:**
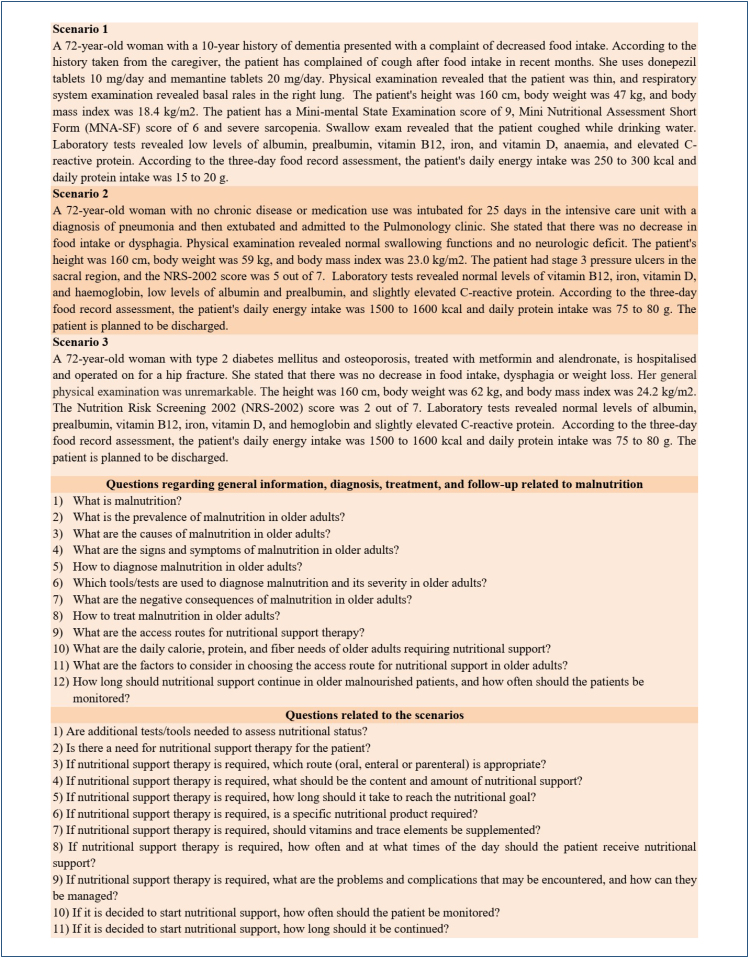
Clinical scenarios and questions related to malnutrition and clinical scenarios.

The study was conducted on 29 June 2025. First, twelve questions regarding general information, diagnosis, treatment, and follow-up related to malnutrition were identified and input into ChatGPT-4o mini. For each question, ChatGPT was asked to respond like a healthcare professional and provide references. To ensure that the results were not affected by previous queries, the browsing history data was completely deleted before each question (Figure 1). Ethics committee approval was not required, as patient data was not used in the present study.

### Clinical scenarios

Three clinical scenarios mimicking real patient information were created (Figure 1).

Scenario 1: A 72-year-old female patient with decreased food intake, difficulty swallowing, advanced dementia, malnutrition, sarcopenia, and clinical findings consistent with aspiration pneumonia.

Scenario 2: A 72-year-old female patient with no chronic illness or medication use who developed pressure ulcers after a long stay in the intensive care unit, with no decrease in food intake and difficulty swallowing.

Scenario 3: A 72-year-old female patient with type 2 diabetes mellitus and osteoporosis who underwent surgery for a hip fracture, with no decrease in food intake and difficulty swallowing.

The scenarios were uploaded to ChatGPT, and the same set of 11 questions was asked to ChatGPT for each scenario (Figure 1). ChatGPT was asked to respond like a healthcare professional to each set of questions and provide references for each answer. Browsing history data was completely deleted before each question set entry.

### Assessment of the responses

ChatGPT's responses were independently examined by three geriatricians. The quality of the responses was assessed using the Quality Analysis of Medical Artificial Intelligence (QAMAI) tool. It consists of six items: accuracy, clarity, relevance, completeness, sources, and usefulness. Each item has a score between 1 (strongly disagree) and 5 (strongly agree), and the sum of the scores yields a total QAMAI score between 6 and 30. The quality grades of the responses are classified as follows based on the total QAMAI score: excellent quality (30 points), very good quality (24–29 points), good quality (18–23 points), fair quality (12–17 points), and poor quality (6–11 points)^13^.

### Statistical analysis

The inter-rater reliability among the three authors was calculated using the intraclass correlation coefficient (ICC). An excellent inter-rater reliability with an ICC of 0.84 (95%CI 0.77–0.89; p<0.001) was calculated. The normality of the data was assessed using the Shapiro-Wilk test. Continuous data are represented as mean±standard deviation. The mean QAMAI scores of the responses generated by ChatGPT were evaluated using the independent samples t-test. The significance level was 0.05, with a confidence interval of 95%. Statistical Package for the Social Sciences (SPSS) for Windows version 22.0 package program was used for statistical analysis.

## RESULTS

The total mean QAMAI score for the ChatGPT-generated responses to questions related to malnutrition was 26.60, indicating very good quality. ChatGPT scored the lowest in the use of sources across all items. In terms of use of sources, responses related to general information and diagnosis had statistically significantly lower scores than responses related to treatment/follow-up (Table 1).

**Table 1:** The Quality Analysis of Medical Artificial Intelligence scores for responses generated by ChatGPT to questions related to malnutrition.

		QAMAI score	p
Accuracy			0.717
	General information	4.70±0.17	
	Diagnosis	4.50±0.50	
	Treatment/follow-up	4.53±0.12	
	Total	4.58±0.29	
Clarity			0.863
	General information	4.63±0.15	
	Diagnosis	4.50±0.50	
	Treatment/follow-up	4.53±0.12	
	Total	4.56±0.27	
Relevance			0.604
	General information	4.63±0.15	
	Diagnosis	4.33±0.58	
	Treatment/follow-up	4.47±0.12	
	Total	4.48±0.33	
Completeness			0.709
	General information	4.50±0.10	
	Diagnosis	4.33±0.58	
	Treatment/follow-up	4.27±0.12	
	Total	4.37±0.32	
Sources			0.012*
	General information	4.05±0.26	
	Diagnosis	3.83±0.29	
	Treatment/follow-up	4.67±0.12*	
	Total	4.18±0.43	
Usefulness			0.520
	General information	4.63±0.15	
	Diagnosis	4.33±0.58	
	Treatment/follow-up	4.33±0.12	
	Total	4.43±0.34	
Total QAMAI score		26.60	

* p<0.05;

the QAMAI scores are presented as mean±SD. QAMAI: Quality Analysis of Medical Artificial Intelligence.

Some of ChatGPT's answers had obvious shortcomings. For example, when asked which tools/tests are used to diagnose malnutrition and determine its severity, it did not mention the Nutritional Risk Screening 2002 (NRS-2002) tool, which is recommended for screening hospitalized patients. In response to the question regarding nutritional needs in older individuals, it was not specified that calorie requirements should be determined based on body weight and disease status. Furthermore, it was not mentioned that protein requirements may increase in the presence of acute and chronic diseases.

In the evaluation of responses to the clinical scenarios, the lowest scores were found to be those related to the use of sources. The total QAMAI score for the clinical scenario involving the patient with a hip fracture was statistically significantly lower than the other scenarios. Accuracy, relevance, and use of sources scores were also statistically significantly lower (Table 2). ChatGPT stated that nutritional support treatment was not immediately necessary for the patient with a hip fracture.

**Table 2:** Comparison of the Quality Analysis of Medical Artificial Intelligence scores of ChatGPT-generated responses to the questions related to clinical scenarios.

	Scenario 1	Scenario 2	Scenario 3	p
Accuracy	4.27±0.52	4.42±0.50	4.00±0.76*	0.048*
Clarity	4.24±0.56	4.45±0.51	4.09±0.74	0.081
Relevance	4.30±0.58	4.48±0.51	4.09±0.70*	0.050*
Completeness	4.21±0.55	4.25±0.44	4.03±0.72	0.379
Sources	4.18±0.53	4.15±0.52	3.70±0.70*	0.008*
Usefulness	4.27±0.52	4.42±0.50	4.03±0.78	0.054
Total QAMAI score	25.48	26.18	23.94*	0.000*

* p<0.05;

the QAMAI scores are presented as mean±SD. Scenario 1, patient with dementia; Scenario 2, patient with pressure ulcers; Scenario 3, patient with a hip fracture. QAMAI: Quality Analysis of Medical Artificial Intelligence.

## DISCUSSION

This study highlights the importance of collaboration between AI technology and healthcare professionals to assess the accuracy of ChatGPT in cases of malnutrition. The overall performance of ChatGPT can be described as very good, as it was able to generate accurate and elaborate responses. A notable limitation was the absence of nutritional support recommendations for specific conditions, along with the lack of adjustment of calorie and protein requirements to disease status and body weight, and the insufficient use of appropriate references.

A recent review evaluating AI and clinical nutrition proposed assessing the clinical efficacy and safety of AI-powered nutrition interventions^8^. AI applications have been developed in many medical specialities, and positive results have been reported. They have the potential to be adapted to clinical nutrition. Nevertheless, it remains essential to ensure that the algorithms employed are transparent, reliable, and clinically validated before they are integrated into routine practice.

ChatGPT's response to the prevalence question on malnutrition indicated that 1 to 10% of community-dwelling older adults are affected by malnutrition. However, it has been shown that the prevalence of malnutrition among older adults worldwide is 18.6%^14^. The lower prevalence of malnutrition reported by ChatGPT may be attributed to limitations in the training data.

When asked which tools/tests are used to diagnose malnutrition and determine its severity, ChatGPT's response was not accurate enough. Because it did not mention the NRS-2002 tool, which is recommended for screening hospitalized patients. The 2024 ESPEN guideline states that amino acids and β-hydroxy-β-methylbutyrate (HMB) can be added to oral/enteral nutrition to accelerate the healing of pressure ulcers, based on the results of randomized controlled trials^15^. In our study, ChatGPT did not mention the use of immunonutrition supplements (arginine, glutamine, and HMB).

Based on the results of our study, ChatGPT has been found to have shortcomings in nutritional support treatment for specific conditions, such as hip fractures. It was stated that nutritional support treatment was not immediately necessary for the patient with a hip fracture. Studies and evidence-based guidelines support the use of oral nutritional supplements (ONSs) for all older patients with hip fractures to reduce nutrition-related complications and enhance clinical outcomes^16,17^. ChatGPT's response may be due to its access to free and publicly available sources, but not to paid, up-to-date sources and guidelines.

In our study, ChatGPT did not specify that calorie and protein requirements should be determined based on body weight and disease status. The reference values for energy intake in older adults are approximately 30 kcal and a minimum of 1 g of protein per kilogram body weight per day, which should be adjusted according to nutritional status, disease status, level of physical activity and tolerability^17^. Additionally, elevated nutritional demands such as those associated with muscle development, tissue repair in cases of malnutrition or wound healing, or heightened metabolic needs during illness should be addressed through a corresponding increase in dietary intake^18^.

The findings of this study reveal that ChatGPT scored the lowest in the use of sources across all items. Although the lack of appropriate references is a limitation, it should be emphasized that a significant portion of ChatGPT's suggestions are consistent with current clinical practice.

A recent study found that integrating an AI-based rapid nutrition diagnostic system into routine in-hospital care significantly improved recovery rates and demonstrated high cost-effectiveness^19^. Machine learning algorithms, by incorporating diverse factors such as age, underlying disease etiology, comorbid conditions, and laboratory parameters, are capable of providing accurate survival predictions while also estimating the potential for improvement in nutritional status. The integration of AI chatbots into routine inpatient care for nutritional deficiencies can make a significant contribution to the medical profession in treating cases of malnutrition.

Our study has some limitations. First, ChatGPT's responses have been evaluated by a relatively small number of experts. Including experts from different fields with experience in nutrition could have increased the assessment's diversity. Second, chatbots other than ChatGPT could also be included to assess their competence in the area of malnutrition and its management. Thirdly, although patients’ body mass index data were available in clinical scenarios, there was no information regarding the amount and duration of weight loss.

Despite these limitations, the study also has some strengths. The areas of knowledge covered by the set of questions were broad, and the quality of the responses was assessed using a validated tool (QAMAI). There was no information in the clinical scenarios that could lead to misunderstanding or misinterpretations, which allowed ChatGPT to be evaluated more objectively.

In conclusion, this study highlights ChatGPT's potential as a valuable tool for providing rapid and accurate information in geriatrics, particularly in the field of clinical nutrition. However, experts emphasized that ChatGPT's answers to some questions were not accurate enough. This was particularly noticeable in its failure to provide dietary recommendations for specific situations, as it did not mention the calorie and protein requirements based on weight and health conditions, and it did not provide adequate references. It is therefore of utmost importance that the responses provided by ChatGPT be critically evaluated and verified. AI-powered decision support systems could assist clinicians in making personalized treatment decisions for patients’ malnutrition.

## Data Availability

The datasets generated and/or analyzed during the current study are available from the corresponding author upon reasonable request.
